# A Cross-Sectional Study of Cannabidiol Users

**DOI:** 10.1089/can.2018.0006

**Published:** 2018-07-01

**Authors:** Jamie Corroon, Joy A. Phillips

**Affiliations:** ^1^ The Center for Medical Cannabis Education, Del Mar, California.; ^2^ Helfgott Research Institute, National University of Natural Medicine (NUNM), Portland, Oregon.; ^3^ Donald P. Shiley BioScience Center, San Diego State University, San Diego, California.

**Keywords:** cannabis, cannabidiol, CBD, marijuana, pain, anxiety

## Abstract

**Introduction:** Preclinical and clinical studies suggest that cannabidiol (CBD) found in Cannabis spp. has broad therapeutic value. CBD products can currently be purchased online, over the counter and at Cannabis-specific dispensaries throughout most of the country, despite the fact that CBD is generally deemed a Schedule I controlled substance by the U.S. Drug Enforcement Administration and renounced as a dietary supplement ingredient by the U.S. Food and Drug Administration. Consumer demand for CBD is high and growing, but few studies have examined the reasons for increasing CBD use.

**Materials and Methods:** A self-selected convenience sample (n = 2409) was recruited via an online survey designed to characterize whom, how, and why individuals are currently using CBD. The anonymous questionnaire was accessed from October 25, 2017 to January 25, 2018. Participants were recruited through social media.

**Results:** Almost 62% of CBD users reported using CBD to treat a medical condition. The top three medical conditions were pain, anxiety, and depression. Almost 36% of respondents reported that CBD treats their medical condition(s) “very well by itself,” while only 4.3% reported “not very well.” One out of every three users reported a nonserious adverse effect. The odds of using CBD to treat a medical condition were 1.44 (95% confidence interval, 1.16–1.79) times greater among nonregular users of Cannabis than among regular users.

**Conclusion:** Consumers are using CBD as a specific therapy for multiple diverse medical conditions—particularly pain, anxiety, depression, and sleep disorders. These data provide a compelling rationale for further research to better understand the therapeutic potential of CBD.

## Introduction

Cannabidiol (CBD) is one of more than a hundred cannabinoids found in *Cannabis sativa* L (*Cannabis* spp. or *Cannabis*), a plant more well known colloquially as marijuana and hemp. CBD is typically the second most abundant cannabinoid found in *Cannabis* after tetrahydrocannabinol (THC).^1^ CBD was first isolated in 1940 and characterized structurally in 1963.^2,3^

CBD is well tolerated in humans and maintains a good safety profile.^4,5^ Neither abuse nor dependence has been demonstrated.^5^ In preclinical studies, CBD shows potential therapeutic efficacy against a diverse assortment of medical conditions. These include seizure disorders, psychotic symptoms, anxiety, depression, inflammation, cancer, cardiovascular diseases, neurodegeneration, symptoms of multiple sclerosis, and chronic pain, either used alone or when coadministered with THC.^5–20^

In October of 2017, a New Drug Application was submitted to the U.S. Food and Drug Administration (FDA) to seek approval of CBD isolated from marijuana for the treatment of two pediatric seizure disorders. Approval was granted in June, 2018, making Epidiolex (cannabidiol) the first plant-derived *Cannabis* compound approved as a drug by the FDA. Availability of Epidiolex is pending Drug Enforcement Administration (DEA) rescheduling of cannabidiol, which is expected to occur within 90 days.^21–23^ Sativex (nabiximols), a combination drug with equal parts CBD and THC extracted from marijuana, is currently approved to treat spasticity due to multiple sclerosis in >30 countries worldwide but is not approved in the United States.^17,21^

The worldwide regulatory status of CBD is complex and constantly changing.^5^ While CBD is legal in many countries as a component of prescription Sativex (nabiximols), it may be simultaneously illegal as a component of a nonapproved *Cannabis* extract containing >0.2% (particularly in European countries) or 0.3% THC. In Europe, individual European Union Member States currently determine the legality of CBD within their borders. Most allow prescription CBD products, as do Australia and New Zealand.^5,24^ Canada became the second nation in the world to legalize *Cannabis* for recreational use in June 2018.^25^ The World Health Organization's Expert Committee on Drug Dependence recommended that CBD should not be controlled by Schedule I of the 1961 UN Single Convention on Narcotic Drugs.^5^ Their comprehensive report is expected this year.

In the United States, until such time as it is rescheduled, CBD from marijuana is deemed by the DEA to fall within the purview of the “marihuana extract rule” (Rule). A dispute over the scope of the Rule was litigated in federal court. The Court found that the Rule applies to extracts of marijuana but that the industrial hemp provisions of the 2014 Farm Act (i.e., “The 2014 Farm Bill”) preempt the Controlled Substances Act (CSA), which the DEA enforces.^26^ Thus, hemp cultivated in compliance with the Farm Bill is not a controlled substance. The Court did not address the issue of CBD directly, however, and left open the issue of the legal status of CBD derived from industrial hemp, from imported “nonpsychoactive hemp” or from parts of the Cannabis sativa plant excluded from the legal definition of marijuana in the Controlled Substances Act of 1970.^27–35^ Despite conflicting legal interpretations, and DEA prohibition, hemp-derived CBD products can currently be purchased as dietary supplements both online and over the counter throughout most of the country. To complicate matters further, the FDA does not recognize CBD as a dietary supplement ingredient because of its status as an Investigational New Drug.^36^

This regulatory confusion has not deterred consumers from exploring the purported benefits of CBD. Retail sales of hemp-derived CBD products in the United States reached $170 million in 2016, and are projected to grow at a 55% compound annual growth rate over the next 5 years to reach >$1 billion. These estimates do not include marijuana-derived CBD.^37^ Although *Cannabis* users have been extensively studied data characterizing the individual use of CBD are scarce. The goal of this study was to collect survey data to elucidate how, and why, individuals are using CBD.

## Methods

### Survey

The study protocol was submitted electronically to the Institutional Review Board (IRB) of San Diego State University. Given the voluntary nature of the survey, and the lack of identifying information, the electronic approval process determined that no IRB approval was necessary.

We developed a novel questionnaire to assess broad characteristics of self-described CBD users and underlying reasons for, and methods of, CBD use. The survey consisted of structured questions answered by either yes/no or multiple-choice responses. Questions focused on several key domains: sociodemographics; reasons for use; duration and frequency of use; method of administration; perceived clinical efficacy; and adverse effects. Study data were collected and managed using Qualtrics^®^ Survey Software, a secure tool allowing participants to directly enter responses anonymously.

Subjects were a self-selected convenience sample who accessed the online survey from October 25, 2017 to January 25, 2018. Recruitment strategies included promotion on survey-specific Web pages on Facebook, LinkedIn, and ResearchGate. CBD product manufacturers and herbal vaporizer manufacturers assisted in recruitment by promoting links to the survey on their Facebook pages and/or via email to their customers. The only inclusion criterion was current or prior use of CBD. Respondents could skip any question(s) they did not wish to answer.

### Data analyses

Descriptive statistics including simple proportions were used to describe demographics, usage characteristics, medical conditions, perceived efficacy, side effects, and other CBD use preferences. Data analyses were conducted using SAS University Edition (SAS 9.4; SAS Institute Inc., Cary, NC). Univariate and bivariate comparisons were conducted using PROC FREQ and chi-square tests. Odds ratios (ORs) were used to estimate strength of association using PROC LOGISTIC. Statistical significance was assessed using α=0.05. Figures were produced using DeltaGraph version 4.5 for Mac.

## Results

### Demographics

A total of 2490 responses were collected. Eighty-one respondents were excluded from the analysis for failure to answer the first question regarding stated use of CBD, leaving 2409 respondents included in the final study population. The sample was balanced in terms of gender (female: 50.87%; male: 47.40%) with most respondents reporting ages between 55 and 74 years (39.97%). Most were either graduates of, or currently enrolled in, college or a postgraduate program (71.22%). The vast majority resided in the United States (91.23%). Respondents from all 50 U.S. states were represented in the survey with the majority residing in California (*n*=412, 21.90%; Table 1). In addition, there were survey respondents from 23 other countries. Regular *Cannabis* use was reported by 55.17% of respondents.

**Table 1.** **Sociodemographic and Other Characteristics of Survey Respondents (*n*=2409)**

	*n* (%)
Gender	
Male	1013 (47.40)
Female	1087 (50.87)
Decline to state	37 (1.73)
Missing	272
Age (years)	
≤24	138 (6.33)
25–34	292 (13.40)
35–44	400 (18.36)
45–54	404 (18.54)
55–64	532 (24.41)
65–74	339 (15.56)
≥75	74 (3.40)
Missing	230
Education	
Primary/middle school	22 (1.01)
High school/GED	503 (23.13)
College	1138 (52.32)
Postgraduate	411 (18.90)
Other	101 (4.64)
Missing	234
Geography	
United States	1987 (91.23)
Canada/Mexico	103 (4.73)
Other	88 (4.04)
Missing	231
Geography—U.S. states (top 5)	
California	412 (21.90)
Texas	93 (4.94)
Oregon	83 (4.41)
Florida	79 (4.20)
Colorado	76 (4.04)
Missing	528
Cannabis use	
Regular	1189 (55.17)
Nonregular	966 (44.83)
Missing	254
CBD use	
General health and well-being	926 (38.44)
Medical condition	1483 (61.56)
Missing	0

CBD, cannabidiol; GED, General Educational Development.

### Stated use: medical versus general health and well-being

More than 60% (61.56%) reported using CBD to treat a medical condition(s) (Table 1). The odds of using CBD to treat a medical condition were 1.65 (95% confidence interval [CI], 1.39–1.97) times greater among women than among men, higher with age, and roughly equal among residents and nonresidents of the United States (OR, 1.06; 95% CI, 0.9–2.5; Table 2). Respondents <18 years of age were subsequently assessed as an independent category despite the small number of observations (*n*=25) and wide CI (OR, 18.72; 95% CI, 4.20–83.39, compared with those between 18 and 24 years of age. Data not included in Table 2). This additional analysis was based on the established use of CBD to treat pediatric seizure disorders^6,7,16^ and the percentage of respondents in that age category reporting using CBD to treat a medical condition (*n*=23; 92%).

**Table 2.** **Odds of Using Cannabidiol for a Medical Condition by Sociodemographic and Other Characteristics (*n*=2409)**

	General health and well-being, (*n*=926) *n* (%)	Medical condition, (*n*=1483) *n* (%)	OR (95% CI)
Gender^***^			
Male	454 (44.82)	559 (55.18)	1.00 (reference)
Female	358 (32.93)	729 (67.07)	1.65 (1.39–1.97)
Decline to state	12 (32.43)	25 (67.57)	1.69 (0.84–3.41)
Missing	102	170	
Age (years)^***^			
≤24	72 (52.17)	66 (47.83)	1.00 (reference)
25–34	153 (52.40)	139 (47.60)	0.99 (0.66–1.49)
35–44	170 (42.50)	230 (57.50)	1.48 (1.00–2.18)
45–54	150 (37.13)	254 (62.87)	1.85 (1.30–2.73)
55–64	180 (33.83)	352 (66.17)	2.13 (1.50–3.12)
65–74	98 (28.91)	241 (71.09)	2.68 (1.80–4.04)
≥75	19 (25.68)	55 (74.32)	3.16 (1.70–5.86)
Missing	84	146	
Education^***^			
College	478 (42.00)	660 (58.00)	1.00 (reference)
Primary/middle school	1 (4.55)	21 (95.45)	15.18 (2.04–113.09)
High school/GED	172 (34.19)	331 (65.81)	1.39 (1.12–1.73)
Postgraduate	153 (37.23)	258 (62.77)	1.22 (0.97–1.54)
Other	34 (33.66)	67 (66.34)	1.43 (0.93–2.19)
Missing	88	146	
Geography			
United States	763 (38.40)	1224 (61.60)	1.00 (reference)
Canada/Mexico/other	76 (39.79)	115 (60.21)	1.06 (0.78–1.44)
Missing	87	144	
Cannabis use^***^			
Regular	509 (42.81)	680 (57.19)	1.00 (reference)
Nonregular	320 (33.13)	646 (66.87)	1.44 (1.16–1.79)
Missing	97	157	

^***^ *p*<0.001.

CI, confidence interval; OR, odds ratio.

The odds of using CBD to treat a medical condition were 1.44 (95% CI, 1.16–1.79) times greater among nonregular users of *Cannabis* when compared with regular users.

### Medical conditions

There were 1483 respondents who reported using CBD to treat at least one medical condition. A minimum of 3963 medical conditions were reported. This represents an average of more than two and a half (mean: 2.67) different medical conditions per respondent.

**FIG. 1.**
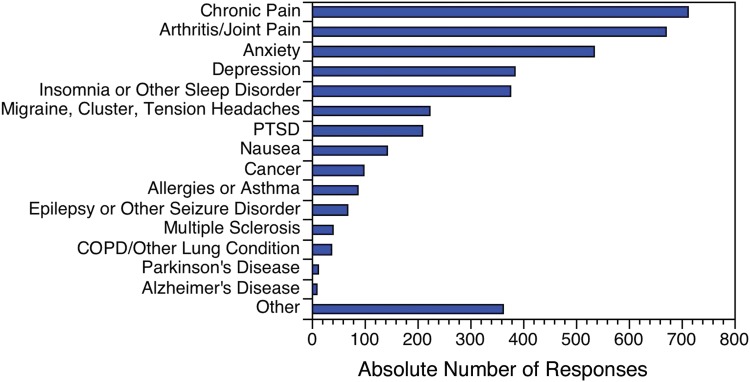
Number of medical conditions for which respondents reported using CBD, by medical condition (*n*=3963). CBD, cannabidiol; COPD, chronic obstructive pulmonary disease; PTSD, post-traumatic stress disorder.

In order of frequency, the top three medical conditions reported were chronic pain, arthritis/joint pain, and anxiety (Fig. 1).

Respondents selected “Other” 362 times. The most common “Other” conditions reported were neuropathy (*n*=48), autoimmune conditions (*n*=38), and fibromyalgia (*n*=37).

### Methods of administration

**FIG. 2.**
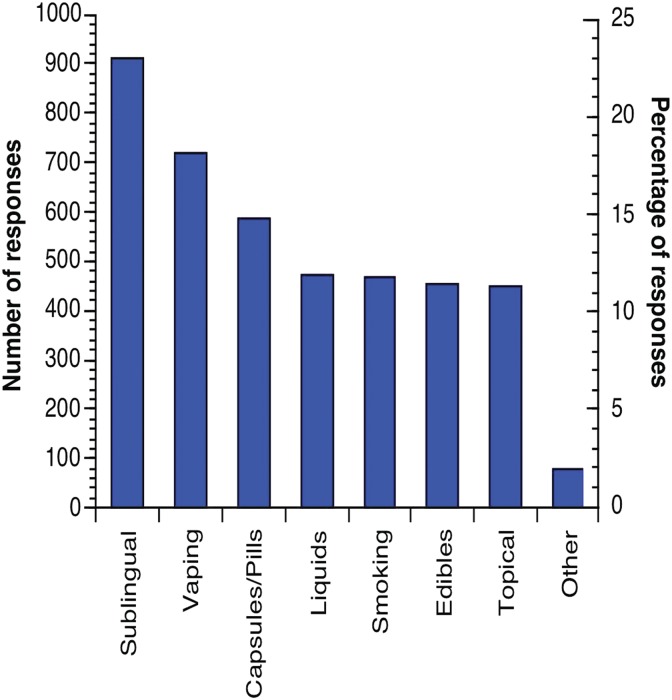
Number and percentage of methods of administering CBD (*n*=4135).

A total of 4135 methods of administration were reported by 2200 respondents. This represents an average of almost two (mean: 1.88) different methods of administration per respondent. Overall, the most common method reported was the administration of CBD in a sublingual form (Fig. 2). This includes liquids administered as sprays, drops, and tinctures. The least common method was topical use.

More than half (51.36%) of those reporting a method of administration reported using only one method (*n*=1130; missing=209; not including “Other,” *n*=31). On average, respondents who reported using one method of administration were 1.6 times more likely to use CBD for a medical condition than for general health and well-being (medical condition, *n*=691; general health and well-being, *n*=439). Medical users reporting one method of administration were 2.4 times more likely to use a topical form, 2.0 times more likely to use an edible form of CBD, and 1.8 times more likely to use CBD in a sublingual or pill or capsule form than general health and well-being users reporting one method.

### Learning about CBD, frequency and duration of use

Overall, 75.85% of respondents reported learning about CBD from internet research, family members, or friends. Of the remaining respondents, medical users were more likely to learn about CBD from a Medical Doctor or Naturopathic Doctor and to use it more frequently than those using it for general health and well-being. The odds of using CBD to treat a medical condition were 1.79 (95% CI, 1.46–2.19) times greater among respondents using it more than once per day compared with those using it daily (Table 3). Duration of use was more variable, but the odds of using CBD to treat a medical condition were greater among those using it for <5 years (Table 3).

**Table 3.** **Odds of Using Cannabidiol for a Medical Condition, by Cannabidiol Usage Characteristics (*n*=2409)**

	General health and well-being, (*n*=926) *n* (%)	Medical condition, (*n*=1483) *n* (%)	OR (95% CI)
Learned about CBD^**^			
Family member/friend	320 (41.24)	456 (58.76)	**1.00 (reference)**
Internet research	337 (38.04)	549 (61.96)	1.14 (0.94–1.39)
Physician/naturopathic doctor	58 (27.36)	154 (72.64)	1.86 (1.33–2.60)
Other (please specify)	131 (41.32)	186 (58.68)	1.00 (0.76–1.30)
Missing	80	138	
Frequency of use^***^			
Daily	418 (39.70)	635 (60.30)	**1.00 (reference)**
<Once per day	227 (56.47)	175 (43.53)	0.51 (0.40–0.64)
>Once per day	196 (26.92)	532 (73.08)	1.79 (1.46–2.19)
Missing	85	141	
Duration of use (years)^*^			
>5	134 (53.17)	118 (46.83)	**1.00 (reference)**
2–5	151 (35.61)	273 (64.39)	2.05 (1.50–2.82)
1–2	202 (42.98)	268 (57.02)	1.51 (1.11–2.05)
<1	364 (34.57)	689 (65.43)	2.15 (1.63–2.84)
Missing	75	135	

^*^ *p*<0.05, ^**^p<0.01, ^***^p<0.001.

### Treatment efficacy

**FIG. 3.**
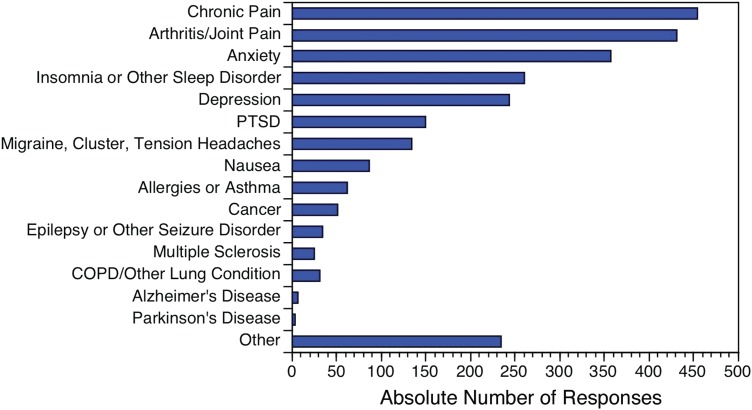
Number of medical conditions for which respondents report CBD treating “Very Well by Itself” or “Moderately Well by Itself,” by medical condition (*n*=2557).

Only respondents who reported using CBD to treat a medical condition were asked about its efficacy (*n*=1483). Almost 36% (35.80%) of respondents reported that CBD treats their medical condition(s) “very well by itself,” while only 4.30% reported “not very well” (Table 4). Respondents most frequently reported feeling that CBD treated their medical condition(s) “very well by itself” or “moderately well by itself” for the following three conditions: chronic pain, arthritis/joint pain, and anxiety (Fig. 3).

**Table 4.** **Number and Percentage of Respondents Using Cannabidiol for a Medical Condition, by Treatment Efficacy and Regular Cannabis Use**

How well do you feel CBD treats your medical condition(s)?	(*n*=1483)	Regular cannabis use (*n*=674) *n* (%)	Nonregular cannabis use (*n*=628) *n* (%)	OR (95% CI)
Not very well	57 (4.30)	18 (31.58)	39 (68.42)	**1.00 (reference)**
Well in combination with conventional medicine^*^	404 (30.44)	195 (49.24)	201 (50.76)	2.11 (1.170–3.82)
Moderately well by itself^***^	391 (29.46)	221 (57.40)	164 (42.60)	2.92 (1.61–5.29)
Very well by itself	475 (35.80)	240 (51.72)	224 (48.28)	2.32 (1.29–4.18)
Missing^**^	156	0	0	0

^*^ *p*<0.05, ^**^p<0.01, ^***^p<0.001.

The odds of regular *Cannabis* use were two to three times greater among those who reported feeling that CBD treated their medical condition “very well by itself” (OR, 2.32; 95% CI, 1.29–4.18) or “moderately well by itself” (OR, 2.92; 95% CI, 1.61–5.29; see Table 4).

### Side effects

A minimum of 1314 side effects were reported across 2409 respondents (missing, *n*=1095). Seven hundred eighty-five (59.74%) of these effects were categorized as adverse (Table 5). On average, this represents at least one reported adverse effect in approximately one out of every three (3.07) users of CBD.

**Table 5.** **Most Common Adverse Effects Reported by Survey Respondents (*n*=742)**

Adverse effect	Medical condition (*n*=1483) n (%)	General health and well-being (*n*=926) n (%)	Total (*n*=2409) n (%)
Dry mouth	174 (11.73%)	94 (10.15%)	268 (11.12%)
Euphoria	59 (3.98%)	96 (10.37%)	155 (6.43%)
Hunger	80 (5.39%)	73 (7.88%)	153 (6.35%)
Other	46 (3.10%)	11 (1.19%)	57 (2.37%)
Red eyes	34 (2.29%)	32 (3.46%)	66 (2.74%)
Sleepy/groggy	29 (1.96%)	14 (1.51%)	43 (1.78%)
Total adverse effects	422 (28.46%)	320 (34.56%)	742 (30.80%)

The top five most frequently reported adverse effects were dry mouth (*n*=268, 11.12% of all CBD users), euphoria (*n*=155, 6.43%), hunger (*n*=153, 6.35%), red eyes (*n*=66, 2.74%), and sedation/fatigue (*n*=43, 1.78%). Just under 30% (28.46%) of medical users reported an adverse effect when compared with 34.56% of general health and well-being users.

## Discussion

To our knowledge, this is the first published survey (aside from industry reports) that specifically analyzes CBD users, as opposed to overall *Cannabis* or medical *Cannabis* users. The results of this study suggest that CBD is used more frequently as a specific therapy for medical conditions than for general health and well-being. This stands in contrast to the majority of marijuana users, who largely use THC-dominant *Cannabis* for recreational or nonmedical reasons.^38^

The most common medical condition for which CBD was reportedly used was pain. In preclinical studies, CBD-based analgesia is associated with potent immune-modulatory, anti-inflammatory, and antioxidant activity.^39–47^ CBD acts as an agonist for a wide variety of cell-surface receptors including adenosine A2_A_, 5-HT_1A_, TRPV1, α7nAch, α3 glycine receptors, and the peroxisome proliferator-activated receptor gamma (PPAR-γ) nuclear receptor. These receptors are all associated with anti-inflammatory activity.^48–58^ Consistent with the efficacy reported by survey respondents, CBD has been shown to reduce inflammatory cytokines in murine models of inflammatory disease and chronic and acute pain.^59^

The endocannabinoid system may also play a role in CBD-mediated analgesia. CBD inhibits enzymes (i.e., fatty acid amide hydrolase and monoacylglycerol lipase) that degrade endocannabinoids. This inhibition is associated with increased endocannabinoid levels, analgesia, and opioid-sparing effects in preclinical models of pain.^60^

Anxiety and depression were also commonly reported reasons for CBD use in this survey. CBD has long been proposed to inhibit THC-associated anxiety by antagonizing cannabinoid receptor activation by THC.^61–63^ CBD may also reduce anxiety via the serotonin 5-HT_1A_ and/or GABA_A_ receptors.^14,64^ These receptor pathways are being explored in hopes of novel therapeutic strategies for phobias, post-traumatic stress disorder, and drug abuse.^65,66^

The majority of survey respondents learned about CBD from internet research, family members, or friends. This was the case for both medical and general health and well-being users. Over 74% of respondents reported using CBD daily or more than daily. Sublingual delivery was the most common route of administration in both groups. The frequency of use of sublingual preparations found in this study contradicts a recent industry-funded CBD survey where respondents reported more frequent vaping, smoking, and topical use.^67^ This industry survey was collected from customers of an online medical marijuana recommendation service. Presumably, these respondents were seeking marijuana-derived products, so the frequency of inhalation as a method of administration is consistent with marijuana users overall.^68^ Our finding may be in part due to the fact that hemp-derived CBD products are largely distributed online and in health-food stores and widely offered as oral preparations.

The percentage of respondents (55.17%) who reported regular *Cannabis* use is markedly higher than national estimates. In 2015, an estimated 8.3% (∼22.2 million people) of individuals aged 12 years or older had used marijuana in the past month.^69^ The reason for the higher rate of *Cannabis* use among survey respondents is not clear, although one possibility is that *Cannabis* users would be more likely to have heard of CBD. However, CBD use by a relatively high percentage (44.83%) of nonregular *Cannabis* users suggests that individuals are not using CBD as a perceived legal route to THC consumption.

Approximately half of all respondents reported using CBD for <1 year. Just over 10% reported using CBD for >5 years. Nonserious adverse effects were relatively common among respondents and higher among those using CBD for general health and well-being, despite the fact that this group reported less frequent use than medical users. While dry mouth, sedation/fatigue, decreased appetite, and diarrhea have previously been reported following CBD use,^6,7^ other studies have demonstrated no adverse effects.^10,70–72^ This dichotomy may be related to dose, interactions with prescription medications, or both. More broadly, adverse effects may also be related to the method of administration and/or the use of purified, high-dose CBD as opposed to CBD in a whole plant extract. These questions and more reinforce the need for more research on unanticipated consequences of CBD use, particularly the impact of long-term usage.^4,5^ Many of the adverse effects reported in this study (i.e., euphoria, hunger, and red eyes) are commonly associated with THC use.^73^ These analyses did not attempt to discriminate between hemp-derived CBD and marijuana-derived CBD products, which may have differing chemical constituents (including THC content) and therefore different effects. Further, no discrimination could be made between isolated CBD and CBD used as a constituent of a whole plant extract.

Industry-originated studies have found that users are confused about the source of their CBD and the concentration of CBD and other ingredients.^67–74^ It is worth noting that independent research has confirmed that the CBD content in almost 70% of CBD-labeled products available online may be mislabeled. In one study, 43% of products were underlabeled and 26% were overlabeled for actual CBD content. More than 20% contained detectable levels of THC.^75^ Since CBD-containing products are largely unregulated there is no obvious way for users to know the quantity of CBD, or other constituents, which may be present in the products they use. Given this uncertainty, it is possible that some of the reported efficacy and the adverse effects may be in part due to the inclusion of other compounds in the CBD preparation, including THC.

### Strengths and limitations

This study has several strengths, including the size, geographic representation of the sample, wide age range of the respondents, and a focus on specific usage characteristics. In part, this was the result of utilizing multiple recruitment methodologies.

In terms of limitations, the study population was a self-selected convenience sample, and as such, may not be representative of the general population or the overall population of CBD users. Individuals with favorable opinions of or experiences with CBD or *Cannabis* are more likely to have responded to the questionnaire than those with negative opinions and experiences. In addition, “regular cannabis use” was not defined in the survey and “marijuana” was not distinguished from “*Cannabis*.” Since the survey was primarily circulated via the internet, CBD users with limited social media connectivity would be underrepresented. Finally, no mechanism for identifying repeat respondents was incorporated into the survey. Although results were examined manually, it is possible that repeat respondents may have distorted the results (i.e., Ballot stuffing).

## Conclusion

The use of CBD among individuals for both specific health conditions and general health and well-being is widespread. The results of this study demonstrate that individuals are learning about CBD from the internet, friends, or family members, rather than from healthcare professionals. CBD is being used as a specific therapy for a number of diverse medical conditions—particularly pain and inflammatory disorders, in addition to anxiety, depression, and sleep disorders. A large percentage of respondents indicate that CBD treats their condition(s) effectively in the absence of conventional medicine and with nonserious adverse effects. These data provide a compelling rationale for further research to better understand the therapeutic potential of CBD in treating chronic pain, anxiety, depression, sleep disorders, and other medical conditions.

## Abbreviations Used

CBD: cannabidiol
CI: confidence interval
COPD: chronic obstructive pulmonary disease
FDA: Food and Drug Administration
GED: General Educational Development
IRB: institutional review board
OR: odds ratio
PTSD: post-traumatic stress disorder
THC: tetrahydrocannabinol
